# Trend Conformity Behavior of Luxury Fashion Products for Chinese Consumers in the Social Media Age: Drivers and Underlying Mechanisms

**DOI:** 10.3390/bs14070521

**Published:** 2024-06-22

**Authors:** Ye Chen, Jingyi Zhuang

**Affiliations:** 1 School of Economics and Management, Zhejiang Sci-Tech University, Hangzhou 310018, China; yechen@zstu.edu.cn; 2 School of Management, Zhejiang University, Hangzhou 310058, China

**Keywords:** luxury fashion product, trend conformity, trend perception, reference group pressure, demand amplification, urge to buy impulsively (UBI)

## Abstract

When a bandwagon consumption trend of luxury fashion products appears, potential consumers tend to conform to the trend. The conformity behavior is enhanced by social media because it makes bandwagon trends more visible. However, no research has explored the drivers of fashion trend conformity in the social media age and the underlying mechanisms. Our empirical research demonstrates that fashion trend conformity is a socially directed type of behavior driven by trend perception and reference group pressure, which represent the informational and normative social influence stimuli, respectively. In addition to the direct impact, we also examine the mediating roles of demand amplification and the urge to buy impulsively (UBI). Demand amplification and UBI, respectively, reflect the rational cognitive reaction and irrational emotional reaction to stimuli of fashion bandwagon consumption. However, our results show that only the cognitive reaction path works, but the emotional reaction path does not. Put simply, trend conformity behavior is largely the result of consumers’ rational reactions rather than irrational reactions to the social influence stimuli of bandwagon consumption. Our study contributes to the research on luxury fashion consumption by introducing three new concepts, i.e., fashion trend conformity, trend perception, and demand amplification, to describe and theorize the characteristics of consumer behavioral patterns for luxury fashion products and new drivers and novel underlying mechanisms of consumer behaviors in the social media age. Our findings offer practical insights for retailers and manufacturers to promote fashion trend conformity behavior.

## 1. Introduction

Luxury fashion products are apparel, accessories, handbags, shoes, watches, jewelry, and perfume characterized by exclusivity, premium prices, image, and status [1,2]. The value of the global fashion luxury market exceeded EUR 280 billion in 2019, of which China accounts for more than one-third [3]. China’s contribution in the global luxury fashion market is predicted to continuously increase [4]. With the phenomenal growth of consumption, exploring what drives consumers to buy luxury fashion products has become a field of growing interest to practitioners and academicians. In addition to self-esteem [5], brand consciousness [6], and the need for uniqueness [7], the need for fashion conformity has been proved to be an important factor determining consumers’ purchase intentions of luxury fashion products, especially in China [8].

Compared with classic luxury products, luxury fashion products are innovative and seasonal (time-sensitive) and usually trigger fashion trends [9]. Thus, we proposed “fashion trend conformity” in this study to distinctively construct conformity behavior in luxury fashion consumption. In social psychology, conformity to consumption trends reflects consumers’ attitudes to follow the convergence of consumption within a group, or even globally [10,11]. The trendy attributes of luxury fashion products may be a more attractive reason for consumers to buy. Consumers are susceptible to fashion trends triggered by luxury fashion products because keeping up with fashion is seen as a form of socializing for people to gain attention from others [12]. Therefore, the most prominent feature of luxury fashion products is bandwagon consumption [13], which refers to the phenomenon that consumers imitate the purchase behavior of a similar majority due to the fear of missing out on luxury fashion trends [14,15]. In our study, fashion trend conformity is defined as an imitative purchase behavior that consumers follow a bandwagon trend of luxury fashion products after observing it. Both bandwagon consumption and trend conformity reflect people’s desire to conform to bandwagon trends. In other words, both belong to consumers’ conformity behaviors. However, compared with “bandwagon consumption” in the extant literature [16,17], trend conformity emphasizes the “visible” role of consumer trends in guiding and promoting conformity consumption behavior in the social media age.

According to social influence theory [18,19], in nature, conformity behavior is the result of social influences, including two basic forms, i.e., informational and normative ones. Previous studies on luxury fashion product consumption have two major shortcomings that reflect the implications of this study to address them. First, no research provides evidence on how informational and normative influences directly drive the consumers’ following behaviors of bandwagon consumption trend for luxury fashion products (or brands). Only one study [20] has found that social influence is related to bandwagon luxury consumption, but not as a driver. The authors investigate the impact of three dimensions of consumers’ need for uniqueness (i.e., creative choice, unpopular choice, and avoidance of similarity) on consumers’ intention for bandwagon and snob luxury consumption, indicating that the creative choice and the avoidance of similarity enhance the purchase intention of bandwagon luxury items indirectly via social comparison. Social comparison refers to a person’s tendency to compare themselves with others in social interactions [21]. Social comparison is a type of social influence similar to normative influence because people obtain both normative and behavioral guidance through comparisons [22]. This study provides inspiration for us to explore the impact of social influences, as drivers, on fashion trend conformity.

Second, no study has focused on the phenomenon of luxury fashion trend conformity (or bandwagon consumption) in the context of social media. In the social media age, individual-to-individual (e.g., consumer-to-consumer) communications on social media platform tend to enlarge the conformity behavior because they make consumption trends more visible [23]. For example, the need for showing off status and the impression of fashion bandwagon leaders drive luxury fashion consumers to post pictures, share reviews, and communicate information on platforms like Facebook, Instagram, and TikTok [24,25]. These consumers’ conspicuous social media contents, compared to the offline showing off of name, front, or logo [16], greatly enhance the visibility of bandwagon consumption trends for luxury fashion products. Due to distinctive attributes of social media, such as fast and wide information dissemination, entertainment, interaction, trendiness, customization, and so on [26], more and more manufacturers, marketers, and retailers are adopting the strategy of social media marketing, including social influencer marketing, to promote positive responses in luxury fashion product consumers. Past research has investigated consumers’ responses to the social media marketing efforts of luxury fashion products (or brands), such as purchase intention [26,27,28,29,30], electronic word-of-mouth (eWOM) [4,24,29], preference [31], price premium [30,31], and loyalty [31]. However, to the best of our knowledge, no research has explored the responses of luxury fashion product (or brand) consumers to social media marketing efforts or content sharing of independent individual users from the perspective of bandwagon consumption or trend conformity.

This study aims to address these shortcomings. Based on the background of social media age, the research questions are as follows: (1) What are the factors driving fashion trend conformity from the perspective of social influence? (2) What is the psychological formation path of fashion trend conformity, that is, what are the mediators between the factors and fashion trend conformity?

To investigate the two research questions, the current study uses social influence theory [18,32] and the stimulus–organism–response (SOR) model [33,34] as theoretical foundations and combines them with the “fear of missing out” (FOMO) model [35,36]. The social influence theory helps us to identify two basic factors to drive fashion trend conformity: fashion trend perception and reference group pressure, which respectively represent informational and normative social influence stimuli. Furthermore, the two factors are enhanced by consumers’ conspicuous behaviors in the context of social media [25]. The SOR model is used to explain the psychological mechanism of how the two factors drive fashion trend conformity. Specifically, we use the SOR model to examine the effects of fashion trend perception and reference group pressure (stimuli) on potential consumers’ fashion trend conformity (response) through demand amplification and the urge to buy impulsively (UBI) (organism), which respectively represent a cognitive reaction path with rational thinking and an emotional reaction path with irrational impulsion to drive the conformity. The FOMO model helps to explain the emotional reaction path of how social influences drive UBI through FOMO and then fashion trend conformity.

This study is organized as follows: Section 2 describes the theoretical background. Section 3 presents the hypotheses’ development. Section 4 explains the research method of the paper. Section 5 presents the data analysis and results. Section 6 presents the discussion and implications for theory and management, and it identifies the limitations of this study and directions for future research. Section 7 presents a concise conclusion.

## 2. Theoretical Background

### 2.1. Social Influence Theory

One outstanding result of social influence is conformity [37]. Conformity refers to aligning one’s behavior or attitude to another person or group [38]. Social influence generally refers to conforming to or identifying with others or the majority [39]. Social influence theory [18] puts forward two forms of social influence: informational and normative social influence. Both social influences lead to conformity, including changes in attitudes, opinions, and behaviors [40,41,42]. Informational social influence refers to the influence of accepting the information of others [18], assuming that others know more about the reality [43]. Therefore, informational conformity is defined as behaving based on the information obtained from others [44]. Normative social influence refers to the influence of complying with the expectations of a group or other people [18], stemming from the need for social identity and returns, such as being accepted or liked by others [45]. Normative conformity refers to a phenomenon that a person’s behavior is influenced by a desire to adhere to conduct standards grounded in a specific group or socially instilled values [46]. Fashion trend conformity is a combined result of these two types of social influence stimuli.

Luxury fashion products are conspicuous consumption goods that can show off consumers’ wealth, social status, taste, and lifestyle [25]. Conspicuous behaviors, especially those of people who potential consumers follow or care about, strongly activate their trend perception of fashion bandwagon and pressure perception of the reference group. Trend perception and reference group pressure reflect the informational and normative social influences of the bandwagon consumption for luxury fashion products, respectively. The two constructs will be discussed further in separate subsections.

### 2.2. SOR Model and FOMO Model

The SOR model (stimulus–organism–response) is a classic psychological model of behavioral science proposed by Mehrabian and Russell in 1974 [33]. This model asserts that environmental stimuli activate an individuals’ affective (emotional) and cognitive reactions, which in turn drive individual behavior [33,34,47]. The extant literature believes that the SOR model can be used to explain the impact of external stimuli on consumer behavior [48,49]. The stimulus refers to the marketing stimulus and/or situational stimulus that arouses consumers [50]. The organism is the affective and cognitive reactions of consumers [51], which are bridges (i.e., mediators) between the marketing and/or situational stimuli and consumer behavioral responses [52]. Finally, the response is consumer purchase decision or behavior [50]. In our study, the response refers to the fashion trend conformity behaviors of potential consumers.

From the perspective of stimulus, social influence theory helps us to identify two basic factors to arouse consumers: trend perception and reference group pressure. Nowadays, social media platforms provide ubiquitous and constant new channels to signal conspicuous consumption [53]. For example, consumers can conveniently post messages about their luxury fashion products through words, pictures, videos, and so forth [25]. The online conspicuous behavior of consumers enlarges the amplitude and frequency of stimuli to potential consumers, thereby driving or enhancing their fashion trend perception and reference group pressure.

From the perspective of the organism, we need to understand the affective and cognitive reaction paths of how trend perception and reference group pressure drive the fashion trend conformity behaviors of potential consumers. Conformity is generally divided into rational conformity and irrational conformity. Rational conformity is a behavior guided by deliberative thinking, judgment, or reasoning [54]. On the contrary, irrational conformity is a behavior guided by intuitionistic and instinctive activities, without deep thinking [54]. For fashion trend conformity, both rational and irrational behaviors likely occur. In the situation of rational conformity, consumers rationally think about and judge their demand of products after being aware of the fashion consumption trend. When going through the process from demand recognition to final purchase, consumers should experience a demand amplification, i.e., an individual cognitive reaction. As a mediator to reflect the cognitive process of fashion trend conformity, demand amplification will be discussed in detail in a separate subsection.

In the situation of irrational conformity, consumers make unplanned purchases without deep thinking or deliberate consideration of alternatives or future implications, involving experiencing an urge to buy impulsively (UBI) [55], i.e., an individual affective/emotional reaction. The fear of missing out on luxury fashion trends is a powerful motivation for bandwagon consumption [14,15]. Hence, the FOMO model is an unavoidable theoretical foundation to explain how social influences drive fashion trend conformity from the perspective of emotional reaction. Specifically, FOMO resulting from social influence stimuli [56] could enhance (UBI), which may lead to fashion trend conformity [36].

FOMO is a significant psychological phenomenon in consumer behavior [56], especially in the context of social media [36]. FOMO is defined as a “pervasive apprehension that others might be having rewarding experiences from which one is absent, … a desire to stay continually connected with what others are doing” [35]. The FOMO definition consists of two basic dimensions: psychological anxiety and the desire for continuous connections with peers. Accordingly, we suggest two dimensions of FOMO in the context of luxury fashion products: the anxiety of being isolated from fashion trends and the desire for belonging to the reference group, which correspond to the two social influences of trend perception and reference group pressure.

Research shows that social motivations drive FOMO [56]. For example, the research of Hayran et al. [57] indicates that FOMO results from social influence because it builds on a comparative evaluation of one’s situation with others. FOMO motivates people to follow trends and not feel left out, as they might regret it [56]. In psychology, FOMO is mostly described as a phenomenon related to affective anxiety and obsessive symptoms, which have been shown to lead to impulsive behavior [36]. In the context of consumption, FOMO could enhance UBI or impulsive purchases [58] because it increases customers’ concerns, which urge their sudden purchases [59]. Often, FOMO drives consumers to impulsively buy products they believe cannot be missed [60]. When more and more consumers have similar behaviors and mutually imitate, conformity consumption behaviors occur [36]. Therefore, the FOMO model explains the emotional reaction path of how social influences drive UBI through FOMO and then fashion trend conformity in the context of luxury fashion products.

**Figure 1:**
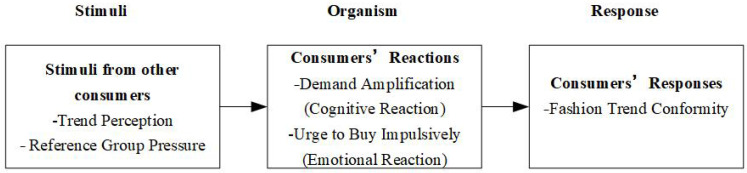
Theoretical framework.

Integrating social influence theory [18] with the SOR model [33], fashion trend perception is identified as a stimulus of informational social influence and reference group pressure is identified as a stimulus of normative social influence. These two stimuli in turn evoke potential consumers’ cognitive reaction (i.e., demand amplification) and emotional (or affective) reaction (i.e., UBI), which will activate their fashion trend conformity behaviors. Due to our research focus on UBI as one of its potential antecedents, FOMO is not introduced in the theoretical framework. The theoretical framework for this study is proposed as shown in Figure 1.

## 3. Hypotheses Development

### 3.1. Fashion Trend Conformity

Fashion trend conformity, as defined above, is a phenomenon that consumers follow the bandwagon trend of luxury fashion products. Social media enlarges this phenomenon because it enables information dissemination to become extremely fast and wide-ranging [61,62]. A large amount of sharing, recommendations, and likes of some luxury fashion products on social media platforms [25] not only promote the formation of fashion trends, but also accelerate and enhance consumers’ perception of fashion trends. Consequently, fashion trend conformity occurs frequently. It is a consumption imitation triggered by bandwagon trends of luxury fashion products. As far as we know, this study is the first to propose the precise concept of fashion trend conformity. Compared with general conformity behavior, fashion trend conformity emphasizes the role of the inherent bandwagon trends of luxury fashion products in leading and promoting conformity consumption behavior. It also considers more about the current social media context, such as the increasing speed of information dissemination and consumers’ rising awareness of fashion. Thus, this concept is more in line with the current consumption patterns and can help to better understand consumer behaviors of luxury fashion products.

### 3.2. Trend Perception

Trend perception is the first basic factor to drive fashion trend conformity, which is identified by social influence theory. A trend refers to the phenomenon of a convergence of the market that results from the mutual imitation of consumers [63]. Research in behavioral science shows that trends are closely related to conformity [33]. Consumers are always attracted by products that have already led the consumer trend, because they believe that the products possessed by the majority are of high quality [64]. Trend perception refers to consumers’ awareness of the popularity of certain commodities based on external information, which implies social recognition and quality reputation [65]. In the context of luxury fashion products, consumers are more sensitive to popular fashion trends due to the fear of missing out [14,15]. Bandwagon consumption is a distinctive phenomenon of luxury fashion products [15]. That is, bandwagon trend perception can drive consumers to make trendy purchases in order to stay in line with most similar consumers. Thus, the following hypothesis is proposed:

**Hypothesis (H1).**  *Trend perception has a positive effect on fashion trend conformity*.

### 3.3. Reference Group Pressure

Reference group pressure is another basic factor to drive fashion trend conformity, which is identified by social influence theory. A reference group refers to a group that an individual uses as a guide for behavior in a specific situation [66]. Reference groups can have a significant impact on one’s attitudes and behaviors [67]. Research shows that consumers’ decisions are strongly influenced by their reference groups [68], especially when the product is a publicly consumed luxury good [69]. Reference group pressure refers to a psychological oppression perceived by a person whose opinions or behaviors conflict with the reference group. The pressure can change people’s behavioral and attitudinal performance to conform to the group [70]. For instance, people often listen to the music preferred by their friends and buy the latest fashionable clothing to help them to fit in [71]. Anspal analyzes the impact of reference groups on the individual consumption of new products, and the results show that reference groups advance the rate of consumer acceptance of trendy products [72]. Indeed, consumers are subject to pressure from the reference group [73].

Although reference group pressure is not a new concept, its impact on consumers’ decisions, especially for luxury fashion products, induces significant change in the context of social media. In the social media age, two reasons make potential consumers of luxury fashion products more susceptible to reference group pressure. First, social media influencers become important members of reference groups. The members are “people” who an individual follows and cares about. They have a key impact on consumer decisions. The members include not only human individuals in real-life interactions, such as family, friends, peers, and celebrities [4], but also virtual individuals in Internet-based interactions, such as online opinion leaders [4] and social media influencers [74,75,76]. Social media influencers are users of social media who have amassed a substantial number of followers through their highly attractive posts or videos [77]. Social media influencers can be either human or virtual/digital [77,78]. By their content sharing about products or brands on social media platforms, both influencers have a powerful impact on consumer purchase intention [77]. Furthermore, compared to human influencers, who have their own commercial considerations and marketing benefits [79], virtual influencers tend to have a higher influence because the latter sharing may be perceived as more trustworthy, credible, and relevant to customers’ preferences [78]. If human or virtual influencers enter the reference group of a potential consumer, their communications on preference for luxury fashion products will mean the potential consumer perceives greater pressure because some other members of the reference group, such as family, friends, and peers, may also be followers of these influencers.

Second, potential consumers of luxury fashion products believe that in the social media age, it is a wise strategy to make product or brand choices by imitating purchase behaviors or accepting the recommendations of reference group members. Before the social media age, the most important information that impacted potential consumers’ decisions was the marketing stimuli of sellers (including retailers and manufacturers), such as advertising and celebrity endorsement through traditional media [80,81]. However, in the social media age, the influence power of traditional marketing is increasingly lost since customers are spending more of their free time on social media [82]. Due to social media’s rapid dissemination of information and the large number of individuals engaged in generating, sharing, and discussing, the impact of social media content about products (or brands) on consumer decisions continuously grows [82]. As audiences of social media content, potential consumers are continuously stimulated by a tremendous number of posts and videos of products generated and disseminated by various users of social media, such as consumers, celebrities, influencers, and sellers. The stimuli of blizzards of other consumers’ conspicuous content sharing toward potential consumers of luxury fashion products are more frequent [25]. An individual does not have enough time, energy, or ability to process the tremendous amount of information. Thus, the best way is to follow the reference group. Furthermore, all members of the reference group are social media users who positively engage in the generation and sharing of content related to luxury fashion products due to conspicuous motivations or commercial considerations, regardless of family, friends, peers, celebrities, online opinion leaders, and human (or virtual) influencers. Their reviews, recommendations, or purchase behaviors for luxury fashion products are visible and can be observed by potential consumers very conveniently.

The members of a reference group may behave in a similar manner [83]. Hence, consumers tend to conform to the group they aspire to be associated with by imitating the purchase behaviors of its major members, which leads to bandwagon consumption [15,16]. In other words, the desire and behavior to keep up with the bandwagon trend will be generated when consumers perceive pressure from the identification and pursuit of a luxury fashion product in a group they care about. Thus, we propose the following hypothesis:

**Hypothesis (H2).**  *Reference group pressure has a positive effect on fashion trend conformity*.

### 3.4. Demand Amplification (Cognitive Reaction Path)

The construct of demand amplification reflects a rational/cognitive reaction path to the stimuli of trend perception and reference group pressure to drive the fashion trend conformity behaviors of potential consumers. Consumer demand can be divided into explicit and implicit demand [84]. Demand amplification refers to the activation or expansion of consumers’ implicit or explicit demands for a commodity, leading to a tension of not owning something. In order to eliminate this tension, purchase motivation is generated, ultimately leading to purchase behavior. In the context of luxury fashion products, demand amplification represents a rational consideration of buying a luxury fashion product when consumers observe the trend of its bandwagon consumption. If a consumer has a motivation to join the bandwagon consumption, it means that she/he sees the utility of that bandwagon product. Product utility is the basis of product demand. However, luxury consumers care more about the symbolic value of a product in social interactions [7,16,85], e.g., conspicuous value of status and wealth [25,86], in addition to the substance value that ordinary goods exclusively emphasize.

The existing literature shows that the consumption demand of luxury products has a positive network externality: one buyer of a commodity increases the value of that commodity to other people [87]. That is, the demand for a luxury commodity is increased due to the fact that others are consuming the same commodity [17]. Obviously, bandwagon consumption is the accumulative result of this externality which, in turn, enhances the externality. Luxury fashion consumption certainly has this positive network externality [87] and a bandwagon will significantly amplify the purchase demands of potential consumers. Since symbolic values underlie the consumption demand for luxury fashion products, we discuss how consumer perceptions of bandwagon consumption drive their identification with the symbolic value and then activate the implicit demand and enlarge the explicit demand to replicate others’ consumption behaviors.

The bandwagon of a luxury fashion product releases several value signals which will promote potential consumers’ demand for that luxury product. First, potential consumers believe that they should buy a bandwagon luxury product because so many others have owned it [16]. Consumers know that the bandwagon luxury product will help them to gain additional utility (i.e., attractiveness) because so many similar others are buying and using them [16,88]. The motivation to get “into the swim of things” and to be “one of the boys” drives demand amplification in two ways. One is to promote the implicit demand for that luxury fashion product to be explicit if consumers have never considered the need for it before. The transformation of demand from implicit to explicit is called the activation of implicit demand. Another is to enhance their explicit demand if they have already had the need for that bandwagon luxury but have not yet determined to buy it immediately.

Second, potential consumers believe that they should buy a bandwagon luxury product immediately because every luxury fashion brand needs to control exclusivity through limited production. Luxury fashion products are “between the mass and the class” mass prestige goods [16]. Although this type of luxury constitutes the lower tier in the luxury pyramid and is priced well below conventional luxury goods, all brands (such as Gucci, Versace, Longchamp, and Luis Vuitton) emphasize that its exclusivity must keep a tenuous balance between top-end luxuries and ordinary premium products [9,16]. When a luxury fashion product shows a bandwagon trend, consumers are aware that its production might be ceased at any time due to limited editions. If consumers do not want to lose the opportunity to chase the current fad and win peers’ identification, they need to realize their demands for the bandwagon luxury as soon as possible by replicating others’ consumption behavior.

Third, potential consumers believe that a bandwagon luxury product deserves its price because so many similar others have identified its symbolic value. Luxury fashion products can satisfy consumer demands for three symbolic values, i.e., uniqueness by moderate limited editions [7], status and wealth signals by moderate–high premium pricing [86], and social identity by triggering the replication of similar others’ consumption behaviors [89]. When a luxury fashion product shows a bandwagon trend, consumers know that so many prior bandwagoners have identified the utility by satisfying the three demands. As long as the price matches with its utility, consumers will be happy to accept the high price for a luxury fashion product instead of feeling that it is so expensive. Naturally, the decreased perception of high price will enhance consumers’ demands for a luxury fashion product.

Taken together, a bandwagon trend will drive potential consumers’ demand for a luxury fashion product in four ways: activating implicit demand, enhancing explicit demand, creating purchase tension, and reducing high-price perception. All four methods of demand amplification tend to drive potential consumers to join the bandwagon, i.e., engage in fashion trend conformity. Therefore, we propose the following hypothesis:

**Hypothesis (H3).**  *Demand amplification has a positive effect on fashion trend conformity*.

In economics, the trend effect refers to the fact that when others’ demand for a good increases in the market, one’s demand for that good will accordingly increase under a given price [17]. The trend effect implies that consumers’ demands are influenced by others’ buying behaviors. In the context of luxury fashion goods, consumers’ purchase demands do not take place in isolation [16] and the market has the typical characteristics of a trend effect. For example, consumers’ demands for a luxury fashion product often originate from attempting to attain peers’ esteem and envy by replicating the consumption of higher social classes [16] when they perceive that a bandwagon has been formed in the mainstream market [17]. As discussed above, bandwagon perception will significantly amplify the purchase demand of potential consumers in four ways. In the social media age, potential consumers can access information about others’ buying behaviors anytime and anywhere because a great number of consumers frequently post conspicuous messages about their luxury fashion products [25]. This will help consumers to perceive the bandwagon of a luxury fashion product, which then amplifies their demands for it. Therefore, the following hypothesis is proposed:

**Hypothesis (H4).**  *Trend perception has a positive effect on demand amplification*.

Consumers strongly desire to have and establish meaningful interpersonal relationships [90]. They construct their desired social identities by making the same choices as others [91]. Since consumers tend to align their views and purchases with others [67,92], their demands for products are susceptible to those people around them or those peers they care about. In the context of luxury fashion consumption, fashion movements are typical representations of normative influence. Potential consumers, who aim to belong to certain reference groups, mirror and identify with their peers and seek to use the same products [93]. If the major members of their reference groups purchase a luxury fashion product, these consumers’ demand for the product will be enhanced. Thus, the following hypothesis is proposed:

**Hypothesis (H5).**  *Reference group pressure has a positive effect on demand amplification*.

### 3.5. Urge to Buy Impulsively (Emotional Reaction Path)

The construct of UBI reflects an irrational/emotional reaction path to stimuli of trend perception and reference group pressure to drive fashion trend conformity. Consumers usually make unplanned purchases spontaneously after being stimulated by price reduction, sales promotion, and limited editions [94]. Such purchases are referred to as impulse purchases, which have three key characteristics: (1) unplanned, (2) result from being stimulated, and (3) decided on the spot [95]. For luxury fashion products, impulse purchase behaviors often take place [96], especially in the context of social media [97,98]. The strong desire to make an unplanned purchase is the urge to buy impulsively [99]. UBI is a sudden and unexpected desire [100], which contributes to the final purchase behavior [101]. It arises before the purchase action and is significantly and positively associated with impulse buying [94,102].

Previous studies show that interactivity, regardless of online context [103,104] or in-store context [105], is an important factor to directly or indirectly drive UBI (or impulsive buying). Interactivity refers to “the degree to which two or more communication parties can act on each other, on the communication medium, and on the messages and the degree to which such influences are synchronized” [106]. Interactivity is the most prominent characteristic of social media because social platforms permit various users, such as consumers, influencers, retailers, and brands, to mutually share experiences, opinions, and information about where, what, and from whom [104,107]. Among social media users, influencers can raise the interactivity to the extreme by responding to followers’ inquiries and providing thoroughly individualized services. Therefore, influencers can let consumers generate various types of emotional attachments to them, which, in turn, drive consumers’ UBI [103]. Obviously, consumers’ UBI is aroused much more easily in the social media age.

Conformity consumption is partially considered as an irrational behavior [54]. As a strong and irrational emotion, UBI can initiate some irrational consumption behaviors. Fashion trend conformity can easily be facilitated by irrational emotion, especially when a luxury fashion product shows a clear bandwagon trend through interactive communications in the context of social media. No research has investigated the impact of luxury fashion characteristics on UBI. We argue that consumers’ fear of missing out on luxury fashion trends [14,15] drives their UBI, which is aroused through four ways: the anxiety of being ignored by the reference group [36]; the anxiety of falling behind and obsessive symptoms of desire for keeping up with the newest fashion trend [36]; the anticipatory pleasure of joining a fashion bandwagon and gaining praise from the reference group [108]; and continuous concerns and interactions [59]. In the context of luxury fashion products, UBI is an emotional reaction of potential consumers to a bandwagon trend, which, in turn, enhances bandwagon consumption. In nature, bandwagon consumption is an extreme conformity because it results from potential consumers’ imitation of others’ purchase behaviors. Thus, we propose the following hypothesis:

**Hypothesis (H6).**  *Urge to buy impulsively has a positive effect on fashion trend conformity*.

Consumers will actively give up controlling themselves when they have certain reasons to do so [109]. Trend perception is a factor that can cause consumers to lose rationality and become impulsive to buy luxury fashion products [110]. Trend perception lets people believe that “what is accepted by many people may also be good for me” [111]. Trend perception drives consumers to imagine how bandwagon consumptions will help them to attain esteem and envy of their fellow people [16]. This is a kind of anticipatory pleasure mentioned above, which can enhance consumers’ UBI. Therefore, we propose the following hypothesis:

**Hypothesis (H7).**  *Trend perception has a positive effect on urge to buy impulsively*.

Prior studies have demonstrated that the reference group has influences on consumer choices [67] for branded products such as perfume [112], wine [113], and clothing [114]. Individuals experiencing negative emotions also likely generates UBI as it can improve their mood and eliminate the discomfort caused by the tension of not owning something [95]. Reference group pressure is precisely a negative emotion that arises when one’s intention conflicts with the reference group [115], thereby evoking UBI. In the social media age, conspicuous behaviors of luxury fashion products ubiquitously happen all the time. These conspicuous behaviors of similar others drive consumers to clearly experience the purchasing pressure from the reference group. Thus, the following hypothesis is proposed:

**Hypothesis (H8).**  *Reference group pressure has a positive effect on urge to buy impulsively*.

UBI is a strong desire to buy when consumers are tempted [99]. The tension of not owning something caused by demand amplification can also trigger a strong desire to purchase immediately, which corresponds to the characteristics of impulse buying [95]. The demand amplification of luxury fashion products results from the stimuli of significant bandwagon trend. Potential consumers’ fear of missing out on luxury fashion trends [14,15] and their anticipatory pleasure of joining a fashion bandwagon will enlarge their demand for luxury fashion products, which, in turn, causes their UBI [116]. Therefore, we propose the following hypothesis:

**Hypothesis (H9).**  *Demand amplification has a positive effect on urge to buy impulsively*.

**Figure 2:**
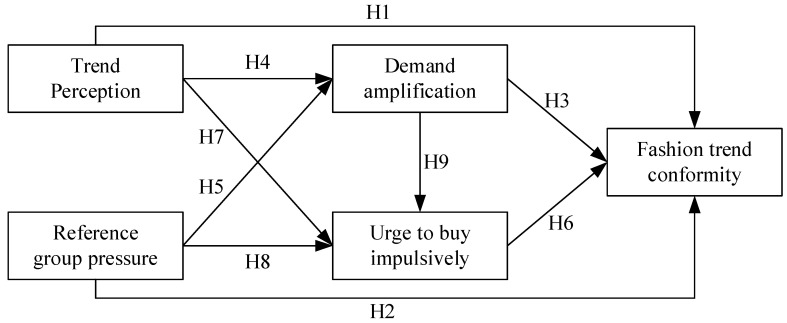
Research model.

The research model is shown in Figure 2.

## 4. Method

### 4.1. Measures

Our research model involves five variables in total, with trend perception and reference group pressure as independent variables, fashion trend conformity as the dependent variable, and demand amplification and the urge to buy impulsively as mediators. Established scales are used to measure reference group pressure, UBI, and fashion trend conformity (see Table 1). The reference group pressure draws on the scale of Bearden and Etzel [67] which is modified to fit the context of the social media age. The scale of UBI is adopted from Beatty and Ferrell [55] and modified to fit the context of luxury fashion consumption. Fashion trend conformity is a new concept in the context of luxury fashion products, but its connotation is similar to that of the “conformity to consumption trend” introduced by Zhou et al.’s study [11]. Its scale is adapted from their scale [11] and modified to fit the context of luxury fashion consumption.

The concept of trend perception, i.e., a perceived bandwagon consumption trend of a luxury fashion product, is relatively new, and there is no mature scale. According to its definition and the context of the social media age, we use two items to reflect the bandwagon connotation (modified from [16]), i.e., “I feel that many people have already purchased the luxury fashion product when seeing ubiquitous online conspicuous behaviors of buyers” and “the luxury fashion product tops the sales charts, which displays a popular trend”, and two items to represent the observation of the popular trend of a luxury fashion product via social media (modified from [27,117]), i.e., “the lifestyle of the luxury fashion product is a leading trend on various social media platforms” and “the luxury fashion product has absolutely consistent positive-reviews”.

Demand amplification is a new concept proposed in this study. By discussing with experts (senior managers and researchers with deep understanding of various luxury fashion industries), a scale with four items is developed, i.e., “my implicit demand for the luxury fashion product is activated when seeing so many people buying it via various digital channels”, “my explicit demand for the luxury fashion product is enlarged when seeing so many people buying it via various digital channels”, “I was not in a hurry to buy the luxury fashion product, but now I can’t wait to buy it when seeing so many people buying it via various digital channels”, and “I thought the luxury fashion product was a bit expensive, but now I suddenly feel that it is not that expensive anymore when seeing so many people buying it via various digital channels”. The measurement items are pre-tested by 52 luxury fashion product users from a major city in eastern China through Wenjuanxing, a professional online survey planform in China, using exploratory factor analysis (pre-tested data are not included in the main analyses). The results of the pilot testing show that the instrument is sound. All items were scored using a 5-point Likert scale (1 = “completely disagree” and 5 = “completely agree”).

The measurement items for all variables are shown in Table 1.

### 4.2. Samples and Data Collection

All of the data in this study were collected online through the professional survey platform Wenjuanxing during December 2023 to January 2024. The links of the recruitment invitation were sent through two social media platforms, WeChat Moments and Sina Weibo. The survey questionnaires were written in Chinese. Using a voluntary response sampling method [118], four hundred and eight Chinese residents participated in the survey. Invalid samples, including those with no purchase experience of luxury fashion products in the last three months, were excluded. The final sample size was 328. According to a general rule [119,120], a sample size between five and ten times the number of items is required. The number of items in this study is 21 (please see Table 1 or Table 2). The sample size of 328 fully meets this criterion and thus can be considered a sufficient number. The sample size is also well above the minimum of 200 observations recommended by Hair et al. (2006) [121] for structural equation modeling analyses.

Among our participants, 69.8% are females and 30.2% were male. Regarding the age of the participants, 76.5% of the sample were aged between 21 and 30 years old, and 23.5% were in other age groups. A majority of the participants were well-educated, with 45.7% holding a bachelor’s degree, 50.3% holding a postgraduate degree, and 14% holding a junior college education or below. The participants’ profiles above are consistent with the stereotypes of the target consumers for luxury fashion products in China: young and well-educated individuals, the majority being women.

In addition, the expense for the last time purchasing of fashion luxury goods was less than RMB 500 (yuan) for 7.6% of participants, between RMB 501 and 2000 for 59.1% of participants, between RMB 2001 and 5000 for 23.8% of participants, and over RMB 5000 for 9.5% of participants. In terms of the expense level for the most recent consumption, the percentage of participants spending over RMB 5000 was low, suggesting that although all the participants can afford some luxury fashion products (light luxury goods), their incomes in general may not be very high.

## 5. Data Analysis and Results

### 5.1. Measurement of Validity and Reliability

Reliability tests of the scales are conducted using SPSS data analysis software 28.0. The results are displayed in Table 2. The Cronbach’s alphas of the scales are all greater than 0.8, reaching the recommended value of 0.6 [122]. The squared multiple correlation coefficients (R^2^) fall between 0.5 and 0.8, indicating that the scales have a good reliability. In addition, the results of the exploratory factor analysis demonstrate that the factor loadings of all items on their corresponding constructs are above 0.5, which tentatively indicates the construct validity and convergent validity of the scales used in this study. In order to further verify the reliability and validity of the scales, confirmatory factor analysis is conducted using the structural equation software AMOS 26.0 (the results are shown in Table 2). The average variance explained (AVE) of each latent variable is greater than 0.5, indicating the convergent validity of the model. The composite reliability (CR) of each latent variable is higher than 0.70, indicating that the scales used in this study have a good indicator reliability.

Discriminant validity test is generally judged by comparing the square root of the average variance explained (AVE) and the absolute value of the correlation coefficient of each latent variable. It can be seen from Table 3 that the square roots of AVEs are greater than 0.5, and the square roots of AVEs of all variables are also greater than the absolute value of the correlation coefficient with other variables. Therefore, there is a good discriminant validity among the variables in this study.

### 5.2. Hypothesis Tests

Confirmatory factor analysis is conducted using AMOS software 26.0, and the test result of the model is shown in Table 4. The ratio of the chi-square value to the degree of freedom (Χ^2^/df) is 1.346. The root mean square error of approximation (RMSEA) is 0.033. The normed fit index (NFI) is 0.949. The comparative fit index (CFI) is 0.986. The goodness-of-fit index (GFI) is 0.941 and the adjusted goodness-of-fit index (AGFI) is 0.915. The value of the Tucker–Lewis index (TLI) is 0.982. All the metrics above indicate that the entire model has a good degree of fitness.

The results show that trend perception (β = 0.119, *p* < 0.05) and reference group pressure (β = 0.148, *p* < 0.05) have a significant positive influence on fashion trend conformity, so H1 and H2 are supported. The stronger the trend perception (β = 0.150, *p* < 0.05) and the reference group pressure (β = 441, *p* < 0.001), the greater their demand amplification; therefore, H4 and H5 hold. Moreover, the stronger the trend perception, the greater the UBI (β = 0.186, *p* < 0.01); H7 holds. In addition, the stronger the demand amplification, the more likely the consumers are to generate UBI (β = 0.567, *p* < 0.001) and fashion trend conformity (β = 0.611, *p* < 0.001); H3 and H9 are valid. However, the effect of reference group pressure on UBI (β = −0.095, *p* > 0.05) and the effect of UBI on fashion trend conformity (β = 0.055, *p* > 0.05) are not significant; thus, H6 and H8 are not verified.

### 5.3. Mediating Effect Tests

We conduct a mediation analysis by Model 4 in PROCESS with 5000 bootstrap samples and 95% bias-corrected intervals [123], and the results are shown in Table 5. We first test the effect of trend perception (TP) on fashion trend conformity (FTC), with demand amplification (DA) and the urge to buy impulsively (UBI) as mediators. The results reveal that the indirect effect of DA is significant since the 95% confidence interval does not include zero, while the indirect effect of UBI is not significant since the 95% confidence interval includes zero. Specifically, the indirect effect of TP on FTC through DA was estimated as 0.2275 (SE = 0.0446, LLCI = 0.1425, ULCI = 0.3171), and the indirect effect through UBI was estimated as −0.0036 (SE = 0.0170, LLCI = −0.0384, ULCI = 0.0294). In addition, the direct effect of TP on FTC was estimated as 0.2058 (SE = 0.0476, LLCI = 0.1121, ULCI = 0.2995) and the total effect was estimated as 0.4297 (SE = 0.0589, LLCI = 0.3138, ULCI = 0.5456). In sum, demand amplification is a partial mediator in the effect of trend perception on fashion trend conformity, but the urge to buy impulsively is not.

As for the effect of reference group pressure (RGP) on FTC, the results show that the indirect effect of DA is significant since the 95% confidence interval does not include zero, while the indirect effect of UBI is not significant since the 95% confidence interval includes zero. Specifically, the indirect effect of RGP on FTC through DA was estimated as 0.2787 (SE = 0.0396, LLCI = 0.2054, ULCI = 0.3585), and the indirect effect through UBI was estimated as 0.0030 (SE = 0.0137, LLCI = −0.0246, ULCI = 0.0311). In addition, the direct effect of RGP on FTC was estimated as 0.1733 (SE = 0.0445, LLCI = 0.0858, ULCI = 0.2607) and the total effect was estimated as 0.4550 (SE = 0.0497, LLCI = 0.3573, ULCI = 0.5527). In sum, demand amplification is a partial mediator in the effect of reference group pressure on fashion trend conformity, while the urge to buy impulsively is not.

## 6. Discussion and Implications

### 6.1. Discussion of Findings

According to the empirical results, both trend perception and reference group pressure are positively related with fashion trend conformity. Trend conformity is the joint result of both informational and normative social influence. Demand amplification mediates the effect, thereby forming an effective cognitive path of fashion trend conformity. However, UBI does not. First, the effect of reference group pressure on UBI is not significant (H8 is not supported). Reference group pressure is more likely to bring negative emotions [115], such as jealousy, low self-esteem, and fear of missing out on the bandwagon. The research of Mick and Demoss points out that UBI has a weak positive relationship with negative emotions [124]. When making a luxury fashion purchase, the negative emotions from reference group pressure may not be strong enough to produce UBI, and therefore there is no significant effect of reference group pressure on UBI.

Second, the influence of UBI on fashion trend conformity is not significant (H6 is not supported). UBI is a strong desire to make an impulsive purchase when facing a highly attractive product. This desire, however, does not necessarily lead to the final purchase behavior, because apart from feeling a strong UBI, consumers may also experience conflict between desire and self-control [99]. Only when the impulse overwhelms self-control will the consumer’s purchase behavior occur. There are also some objective factors which may prevent UBI from leading to fashion trend conformity, such as the price of products. We argue that people likely display higher self-control when facing high-price premium products without a sufficiently slack budget. Most participants in this study are young people with ages from 21 to 30, whose income may not be high enough to allow slack budgets. For those products with an unaffordable price, these potential consumers can calm down and think about the necessity of their demand. They are bound to give up if their true demands are not strong enough.

The invalid mediating effect of UBI reveals that trend perception and reference group pressure cannot influence trend conformity through an irrational emotional path. In contrast, demand amplification, which represents a rational cognition path, is an effective mediator. Taken together, in the context of luxury fashion products, consumption rationality triumphs over consumption irrationality. Therefore, to a greater extent, fashion trend conformity can be regarded as a rational, rather than an irrational, behavior, which implies that consumers make decisions based on considerations of utility and demand for luxury fashion products.

### 6.2. Theoretical Implications

Our study extends the research on the consumption of luxury fashion products in several ways. First, we propose the concept of fashion trend conformity to construct the specific conformity behavior of luxury fashion consumption. Fashion trend conformity precisely describes the consumption imitation phenomena triggered by consumer trends. Compared with “bandwagon consumption” in the extant literature [16,17], which also implicitly reflects consumer conformity behavior, trend conformity emphasizes the “visible” role of consumer trends in guiding and promoting conformity consumption behavior in the social media age. The construct of fashion trend conformity not only captures the impact of the “fad chasing” characteristics of luxury fashion consumption on consumer decisions, but also resonates with the current social media context, which makes consumer trends more visible. This concept intuitively reflects the formation principle of the luxury fashion bandwagon effect, i.e., a consumer trend activates the consumption imitation of potential consumers, which in turn enlarges the consumer trend. Put simply, in the context of luxury fashion products, bandwagon consumption trends interactively and repeatedly drive consumer conformity. Overall, this concept is more in line with the current consumption patterns of luxury fashion products in the social media age and is useful for theoretical and practical research on consumer behaviors.

Second, we reveal what drives fashion trend conformity by jointly adopting the theory of social influence and the SOR model. Our findings show that trend perception and reference group pressure are two fundamental factors which determine potential consumers’ conformity behaviors. The two factors, respectively, reflect the informational and normative social influences which stem from the stimuli of fashion bandwagon trends. The trend perception and reference group pressure for potential consumers have become more significant in the social media age. The reason is not hard to understand, i.e., consumers’ ubiquitous conspicuous behaviors of fashion bandwagon consumption on various social media platforms. No research on luxury bandwagon consumption has investigated these two basic drivers in the social media age.

Third, we reveal the rational and irrational psychological mechanisms that underlie consumers’ trend conformity behaviors of luxury fashion products. According to the SOR model, we examine two psychological mechanisms, i.e., demand amplification and UBI (the urge to buy impulsively), which reflect the cognitive and affective reactions of potential consumers to the stimuli of luxury fashion bandwagon trends, respectively. Our findings show that demand amplification is a partial mediator between trend perception and reference group pressure and fashion trend conformity, but UBI has no significant mediating effect. These insights mean that the conformity behaviors of luxury fashion products are rational reactions, rather than irrational reactions, to the social influence stimuli of fashion bandwagon consumption. We put forward “demand amplification” for the first time, which reflects the rational thinking driving consumers’ trend conformity behaviors of luxury fashion products. Demand is the anchor of purchase decisions. Luxury consumption is expensive. Consumers will not give up rational demand considerations when making purchase decisions, even in their seemingly irrational “fad chasing” behaviors. Our insight into the impact of demand amplification on fashion trend conformity helps to open the black box of the formation process of luxury fashion bandwagon consumption.

### 6.3. Managerial Implications

Our study has two managerial implications. First, luxury retailers and manufacturers could foster consumers’ trend conformity by enhancing their fashion trend perception and reference group pressure, which represent the informational and normative stimuli of the bandwagon consumption of luxury fashion products, respectively. Overall, our empirical results show that the trend conformity of luxury fashion product is a socially directed type of behavior that is driven by trend perception and reference group pressure. As consumers are inclined to follow consumer trends due to the fear of missing out on fashion trends, creating the sense of a trend for products by social media marketing is a good strategy to foster conformity consumption. Since consumers are susceptible to reference group pressure, encouraging consumers to convey a group expectation by sharing or recommending products to their friends is also an effective way to activate consumer conformity. Specifically, luxury retailers and manufacturers could create brand communities on social media platforms to incentivize consumers to post their luxury fashion product purchases and usage experiences, share buyers reviews and purchase advice, and post luxury fashion vlogs.

Keeping customers informed of the latest luxury fashion products or events is key to the fashion trend perception of potential consumers. Luxury retailers and manufacturers could also associate their brands with the identity of certain groups. Meanwhile, managers should help consumers to interact with their preferred groups. The interactions among consumers allow them to feel mutual identification with each other via chasing the same fashion trends. If potential consumers frequently interact with these groups, they will generate pressure or a strong need to join the trends as soon as possible by conforming to the consumption behaviors.

Second, our study helps luxury retailers and manufacturers to realize that, to a great extent, fashion trend conformity is a rational consumption behavior. Our empirical results show that, in addition to a direct impact, trend perception and reference group pressure also indirectly influence fashion trend conformity via some psychological mechanisms. We predict that demand amplification and UBI (the urge to buy impulsively) will be mediators between trend perception and reference group pressure and fashion trend conformity. Demand amplification and UBI, respectively, reflect cognitive reactions and emotional reactions to fashion bandwagon consumption stimuli, corresponding to rational and irrational consumption psychological mechanisms. Our study confirms that the cognitive reaction path works, but the emotional reaction path does not. These findings indicate that trend conformity behaviors are primarily the results of consumers’ rational reactions rather than irrational reactions to bandwagon consumption stimuli. Consumers imitate others’ purchase behaviors because the bandwagon consumption stimuli activate their implicit demands and enlarge their explicit demands. These insights are consistent with the characteristic of luxury fashion products with a high premium, which drives consumers to make purchase decisions based on utility. The extant literature (e.g., [16,17] manifests that the utility of luxury fashion products mainly originates from their symbolism value. Thus, retailers and manufacturers should encourage consumers in brand communities to share stories about how the bandwagon consumption of luxury fashion products satisfies their demands for uniqueness, status, wealth, social identity, etc. When seeing so many people buying a luxury fashion product and telling their stories, it seems almost inevitable that potential consumers will amplify their demands for the bandwagon product.

### 6.4. Limitations and Future Research

Our study has some limitations that suggest possibilities for future research. First, the mediating role of UBI on fashion trend conformity is not proved, indicating that the irrational psychological mechanism of the emotional reaction path does not work in our empirical context. The average income of the respondents in this research is not very high. Our study does not investigate the moderation role of income level, price level, and their matching relationship. If spending budgets do not allow them to rashly make unplanned purchases of high-price premium products, consumers will do their best to control their urge to buy impulsively. In fact, however, irrational behavior does exist in luxury fashion consumption [96]. Future researchers can explore the trend conformity behavior of consumer groups with different levels of income for luxury fashion categories with different levels of prices.

Second, our study validates the mediating effect of demand amplification on fashion trend conformity; however, it does not explore the symbolic utilities which underlie the demand implication of potential consumers, which stem from joining fashion bandwagon trends. Future research can explore the impacts of utility variables on demand amplification from multiple perspectives of symbolism values, for example, the symbols of uniqueness, status, wealth, image, and social identity.

Third, the generalizability of the findings may be limited due to sample representativeness. Participants were recruited using a voluntary response sampling method [118] because it is difficult to adopt a random sampling method in the survey of consumer behaviors. Although the sample size was sufficiently large in this study, the possible bias of a voluntary sample could limit the generalizability of the findings [78,118]. In addition, the participants were only from China. Chinese consumers have a typical collectivistic culture orientation, which drives individuals’ conformity behaviors more easily compared with an individualistic culture orientation. Future research could explore the impact of culture differences on consumers’ trend conformity behaviors in buying luxury fashion products.

## 7. Conclusions

The most prominent feature of luxury fashion products is bandwagon consumption, which drives the conformity purchases of potential consumers due to their fears of missing out on the bandwagon trends. The powerful stimuli of consumers’ conspicuous content sharing flooding social media platforms enhance the fashion trend conformity behaviors of potential consumers. Using social influence theory and the SOR model jointly, we explore the drivers of fashion trend conformity in the social media age and the underlying mechanisms. Specifically, we examine the effects of fashion trend perception and reference group pressure (stimuli) on potential consumers’ fashion trend conformity (response) through demand amplification and the urge to buy impulsively (UBI) (organism). Trend perception and reference group pressure reflect the stimuli of informational and normative social influences, respectively. Demand amplification and UBI, respectively, represent a cognitive reaction path with rational thinking and an emotional reaction path with irrational impulsion to drive the conformity.

Our empirical evidences show that fashion trend conformity is a result driven by the stimuli of trend perception and reference group pressure. However, only the cognitive reaction path works, but the emotional reaction path does not. Put simply, the trend conformity behaviors of luxury fashion products are largely the result of consumer’s rational reactions rather than irrational reactions to the social influence stimuli of bandwagon consumption.

In our study, we put forward three new concepts: fashion trend conformity, demand amplification, and trend perception. Combined with reference group pressure and UBI, these five constructs describe and theorize the characteristics of consumer behavioral patterns for luxury fashion products, behavioral drivers, and underlying mechanisms in the social media age.

## Figures and Tables

**Table 1:** Measurement items used.

Variable	Items	Question
Trend Perception (TP)	TP1	I feel that many people have already purchased the luxury fashion product when seeing ubiquitous online conspicuous behaviors of buyers.
	TP2	The luxury fashion product tops the online sales charts, which displays a popular trend.
	TP3	The lifestyle of the luxury fashion product is a leading trend on various social media platforms.
	TP4	The luxury fashion product has absolutely consistent positive reviews.
Reference Group Pressure (RGP)	RGP1	People who I follow or care about have already purchased the luxury fashion product.
	RGP2	People who I follow or care about recommended the luxury fashion product via public and private digital channels.
	RGP3	People who I follow or care about thought the luxury fashion product should be purchased to keep up with the newest fashion trend.
	RGP4	People who I follow or care about have shared information of the luxury fashion product.
	RGP5	Most of the people who I follow or care about have given the luxury fashion product positive reviews.
Demand Amplification (DA)	DA1	My implicit demand for the luxury fashion product is activated when seeing so many people buying it via various digital channels.
	DA2	My explicit demand for the luxury fashion product is enlarged when seeing so many people buying it via various digital channels.
	DA3	I was not in a hurry to buy the luxury fashion product, but now I can’t wait to buy it when seeing so many people buying it via various digital channels.
	DA4	I thought the luxury fashion product was a bit expensive, but now I suddenly feel that it is not that expensive anymore when seeing so many people buying it via various digital channels.
Urge to Buy Impulsively (UBI)	UBI1	I had the sudden urge to purchase the luxury fashion product outside my shopping list.
	UBI2	I experienced a number of sudden urges to buy the luxury fashion product I had not planned to purchase.
	UBI3	I had the inclination to buy the luxury fashion product although I know that it is outside my shopping goal.
	UBI4	I thought the luxury fashion product was ordinary, but suddenly I thought it was very good.
Fashion trend conformity (FTC)	FTC1	I bought the luxury fashion product that firmly made me feel good in my social group.
	FTC2	I bought the luxury fashion product that firmly gave me the sense of fashionable and stylish belonging.
	FTC3	I bought the luxury fashion product that firmly causes me to make a good impression on others.
	FTC4	I bought the luxury fashion product that firmly makes me feel a part of the fashion trend.

**Table 2:** Construct reliability and validity.

Variable	Item Number	Cronbach’s α	CR	AVE
Trend perception	4	0.848	0.846	0.581
Reference group pressure	5	0.880	0.890	0.669
Urge to buy impulsively	4	0.843	0.838	0.567
Demand amplification	4	0.878	0.877	0.641
Fashion trend conformity	4	0.865	0.861	0.609

**Table 3:** Test results of discriminant validity.

Variable	Trend Perception	Reference Group Pressure	UBI	Demand Amplification	Fashion Trend Conformity
Trend perception	0.762				
Reference group pressure	0.456	0.818			
Urge to buy impulsively	0.272	0.255	0.753		
Demand amplification	0.310	0.458	0.520	0.801	
Fashion trend conformity	0.375	0.452	0.370	0.690	0.780

Note: The value on the diagonal is the square roots of the AVEs. The value under the diagonal is the absolute value of the correlation coefficient of each latent variable.

**Table 4:** Test results of the model.

Construct Relation	Standardized Path Coefficient	S.E.	C.R.	*p*	Hypothesis Test
TP → FTC	0.119 *	0.090	2.096	0.036	H1 holds
RGP → FTC	0.148 *	0.073	2.471	0.013	H2 holds
DA → FTC	0.611 ***	0.066	8.221	0.001	H3 holds
TP → DA	0.140 *	0.125	1.986	0.047	H4 holds
RGP → DA	0.441 ***	0.101	6.010	0.001	H5 holds
TP → UBI	0.186 **	0.088	2.618	0.009	H7 holds
RGP → UBI	−0.095	0.071	−1.289	0.197	H8 not hold
DA → UBI	0.567 ***	0.057	6.952	0.001	H9 holds
UBI → FTC	0.055	0.080	0.875	0.381	H6 not hold
Model Fitting Metrics	Χ^2^/df = 1.355; RMSEA = 0.034; GFI = 0.941; AGFI = 0.915; CFI = 0.986; TLI = 0.982; NFI = 0.949				

Note: S.E. is the standard error of the estimates. C.R. is the critical ratio. * *p* < 0.05, ** *p* < 0.01, *** *p* < 0.001.

**Table 5:** Mediating effects.

**Effect of TP on FTC**					
		**Effect**	**SE**	**LLCI**	**ULCI**
Indirect effect	TP→DA→FTC	0.2275	0.0446	0.1425	0.3171
	TP→UBI→FTC	−0.0036	0.0170	−0.0384	0.0294
Direct effect	TP→FTC	0.2058	0.0476	0.1121	0.2995
Total effect	TP→FTC	0.4297	0.0589	0.3138	0.5456
**Effect of RGP on FTC**					
		**Effect**	**SE**	**LLCI**	**ULCI**
Indirect effect	RGP→DA→FTC	0.2787	0.0396	0.2054	0.3585
	RGP→UBI→FTC	0.0030	0.0137	−0.0246	0.0311
Direct effect	RGP→FTC	0.1733	0.0445	0.0858	0.2607
Total effect	RGP→FTC	0.4550	0.0497	0.3573	0.5527

Abbreviations: TP, trend perception; RGP, reference group pressure; DA, demand amplification; UBI, urge to buy impulsively; FTC, fashion trend conformity.

## Data Availability

The data supporting the findings of this study are available from the corresponding author upon reasonable request.
